# Walk on the wild side: SIV infection in African non-human primate hosts—from the field to the laboratory

**DOI:** 10.3389/fimmu.2022.1060985

**Published:** 2023-01-12

**Authors:** Anna J. Jasinska, Cristian Apetrei, Ivona Pandrea

**Affiliations:** ^1^ Division of Infectious Diseases, Department of Medicine (DOM), School of Medicine, University of Pittsburgh, Pittsburgh, PA, United States; ^2^ Department of Infectious Diseases and Immunology, Graduate School of Public Health, University of Pittsburgh, Pittsburgh, PA, United States; ^3^ Department of Pathology, School of Medicine, University of Pittsburgh, Pittsburgh, PA, United States

**Keywords:** AIDS - acquired immunodeficiency syndrome, HIV - human immunodeficiency virus, nonhuman primate (NHP), African green monkey (AGM) (Chlorocebus aethiops), mucosal immune barrier, inflammation, microbiome, immune activation

## Abstract

HIV emerged following cross-species transmissions of simian immunodeficiency viruses (SIVs) that naturally infect non-human primates (NHPs) from Africa. While HIV replication and CD4^+^ T-cell depletion lead to increased gut permeability, microbial translocation, chronic immune activation, and systemic inflammation, the natural hosts of SIVs generally avoid these deleterious consequences when infected with their species-specific SIVs and do not progress to AIDS despite persistent lifelong high viremia due to long-term coevolution with their SIV pathogens. The benign course of natural SIV infection in the natural hosts is in stark contrast to the experimental SIV infection of Asian macaques, which progresses to simian AIDS. The mechanisms of non-pathogenic SIV infections are studied mainly in African green monkeys, sooty mangabeys, and mandrills, while progressing SIV infection is experimentally modeled in macaques: rhesus macaques, pigtailed macaques, and cynomolgus macaques. Here, we focus on the distinctive features of SIV infection in natural hosts, particularly (1): the superior healing properties of the intestinal mucosa, which enable them to maintain the integrity of the gut barrier and prevent microbial translocation, thus avoiding excessive/pathologic immune activation and inflammation usually perpetrated by the leaking of the microbial products into the circulation; (2) the gut microbiome, the disruption of which is an important factor in some inflammatory diseases, yet not completely understood in the course of lentiviral infection; (3) cell population shifts resulting in target cell restriction (downregulation of CD4 or CCR5 surface molecules that bind to SIV), control of viral replication in the lymph nodes (expansion of natural killer cells), and anti-inflammatory effects in the gut (NKG2a/c^+^ CD8^+^ T cells); and (4) the genes and biological pathways that can shape genetic adaptations to viral pathogens and are associated with the non-pathogenic outcome of the natural SIV infection. Deciphering the protective mechanisms against SIV disease progression to immunodeficiency, which have been established through long-term coevolution between the natural hosts and their species-specific SIVs, may prompt the development of novel therapeutic interventions, such as drugs that can control gut inflammation, enhance gut healing capacities, or modulate the gut microbiome. These developments can go beyond HIV infection and open up large avenues for correcting gut damage, which is common in many diseases.

## 1 Introduction

The statement “The origin of AIDS is more ancient than the origin of HIV-1” by Sharp and Hahn (1) emphasizes the long-term presence of the SIVs, the simian ancestors of the HIV pandemic strains, in African non-human primate (NHP) populations (2–7). Species-specific strains of SIV have emerged and spread among African NHPs through the codivergence of SIV lineages with host speciation, e.g., contemporarily, SIVs are naturally infecting over 45 NHP species in Africa, while they are absent in NHPs native to the Asian continent and the Americas (5, 8, 9).

HIV-1 emerged through cross-species transmission of SIV to humans (1), leading to massive CD4^+^ T-cell loss, inflammation, and chronic immune activation in the gut and systemically, which altogether eventually lead to an exhaustion of the immune system and the development of opportunistic infections and AIDS-associated comorbidities, such as hypercoagulation and other cardiovascular diseases, hyperlipidemia, chronic kidney and hepatic diseases, osteoporosis, endocrine diseases, and cancers (10, 11). While some aspects of AIDS/HIV pathogenesis are reproduced in experimentally infected Asian macaques that are vulnerable to SIVmac-induced immunodeficiency (12, 13), the vast majority of modern-day African NHPs manifest a benign course of natural SIV infection when infected with their species-specific SIV strains (14–20). These natural host species do not progress to immunodeficiency despite life-long viremia and CD4^+^ T-cell loss in the gut and periphery, e.g., sooty mangabey (SM, *Cercocebus atys*) (18, 21), African green monkey (AGM, genus *Chlorocebus*) (22, 23), and mandrills (MND, *Mandrillus sphinx*) (19, 24).

Although the course of SIV infection in the natural host species is generally benign, several lines of evidence demonstrate that the viruses that naturally infect African NHP hosts do, in fact, retain their virulence and have the capacity to cause pathogenic infections; however, this is controlled by the superior species-specific host defense mechanisms (1). Similar features of SIV infections between natural hosts and progressing hosts (25–27) include early and massive loss of CD4^+^ T cells in the gut mucosa (23, 28–30) but the ability to preserve healthy levels of Th17 cells, which play a key role in mucosal immunity (promoting the recruitment of neutrophils and expression of antimicrobial products) (31, 32), in contrast to SIV-infected macaques (32) and HIV-infected humans (31) (2). The description of rare cases of AIDS in SIV-infected African NHPs after a long incubation period or in senescing individuals revealing that the pathogenic effects of SIV manifested in a background of a weakening immune system (33, 34): a feral born AGM (aged 12 years) (35), a captive-born SM naturally infected by SIVsmm transmitted in the colony (aged 20 years) (36), a feral-born mandrill naturally infected in the wild (aged 18 years) (37), and a black mangabey experimentally infected with SIVsmm (38) (3). Pathogenicity of species-specific strains from natural hosts to at least some species of Asian macaques upon direct cross-species transmission is routinely leveraged by employing the rhesus macaque (*Macaca mulatta*), the pigtailed macaque (*Macaca nemestrina*), and the cynomolgus macaque (*Macaca fascicularis*) as surrogate models for studying HIV-1 infection in humans (39, 40) (4). Lack of immunodeficiency or other disease in AGMs infected with a pathogenically enhanced HIV-1-like SIVagm (expressing Vpu and Nef proteins) was reported over a 5-year infection/follow-up. Despite being associated with moderately increased levels of chronic immune activation, this infection neither accelerated CD4^+^ T-cell depletion nor caused overt AIDS (41). Taken together, the general lack of disease progression in the natural hosts is not due to differences in natural history or to a lack of cytopathicity of the virus but rather the result of active protective mechanisms against immunodeficiency resulting from host adaptations to highly replicating virus.

**Figure 1:**
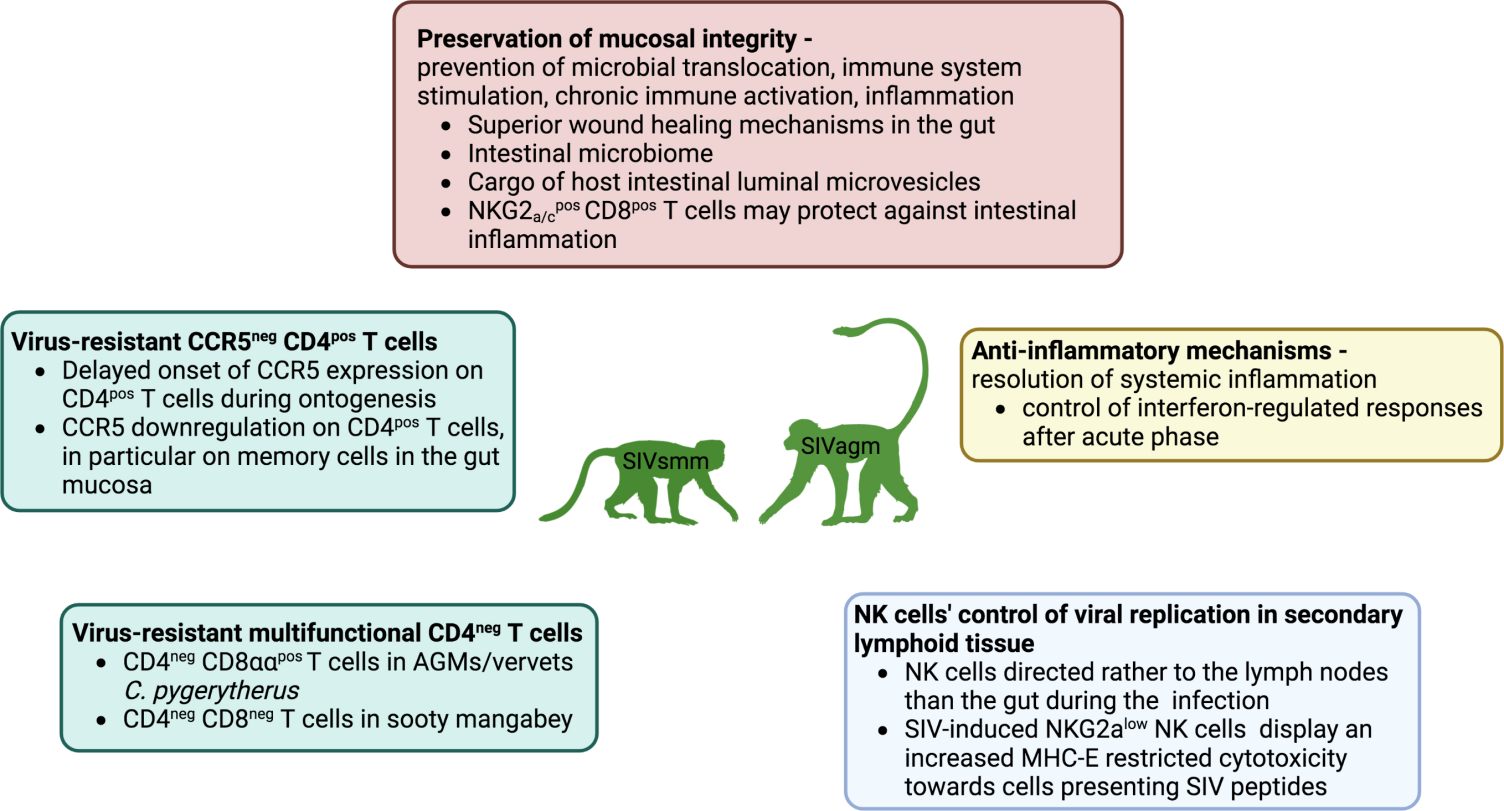
Mechanisms of protection against SIV infections include homeostatic regulation of immune cell populations through target cell restriction by downregulation of CD4 or CCR5 in T-cells (green frame) and NK cell colocalization in secondary lymphoid tissues, expansion and enhanced MHC-E restricted activity (blue frame), preservation of mucosal immunity, and thus prevention of microbial translocation, immune system stimulation and chronic immune activation. Inflammation is undergirded by highly effective non-inflammatory wound healing in the intestinal mucosa, the anti-viral content of epithelial microvesicles in the gut, and a potential anti-inflammatory role of NKG2a/c^+^CD8^+^ T cells in the gut, while the role of the intestinal microbiome remains unclear (red frame), and systemic anti-inflammatory mechanisms control the expression of interferon-stimulated genes after the acute phase (yellow).

This ‘well-tempered SIV infection’ (42) in the African NHP hosts has been previously linked to several main protective strategies (1): a lack of microbial translocation from the gut, and therefore, a lack of stimulation of inflammation and immune activation (43) (2); anti-inflammatory mechanisms controlling these processes and preventing chronic inflammation (44–46) (3); homeostatic regulation of various immune cell populations, including the sparing of critical immune cell subsets through the downregulation of CD4 receptors (47–50) while preserving some of their functionalities, or downregulation of CCR5 co-receptor on the CD4^+^ T-cell surface at sites critical for SIV pathogenesis (51) and in immature individuals (52–54) (**Figure 1**).

The identification of critical differences in the host mechanisms counteracting the pathogenic HIV infection in humans (or SIV infection of experimental NHP models of simian AIDS) and non-pathogenic infection in some natural hosts may shed light on the host mechanisms controlling the pathogenicity of lentiviral infection and inspire novel approaches to HIV/AIDS treatment.

## 2 The natural hosts of SIVs are the origin of pathogenic SIVs and HIVs

SIV is an old pathogen in NHPs, as suggested by the lack of pathogenicity in the host species and demonstrated by two studies that combined molecular phylogeny with biogeography to recalibrate molecular clock analyses. The first study compared SIV sequences originating from the same species collected from Bioko Island and the Equatorial Guinea African mainland, which were once connected *via* a land bridge but became separated 12,800 years ago at the end of ice age. Recalibration of the molecular clocks to include the biogeography, and thus the fact that the last time when these two strains could have been in contact was before the collapse of the land bridge, led to the conclusion that SIV could have been infecting these NHP species for at least 32,000 years but was likely much more ancient (4).

The second study was performed on vervets from South Africa, which have been postulated to have coevolved with SIV over a long period of evolutionary time, initially estimated to be between 3 million to 100,000 years ago, with limits corresponding to the vervets dispersal across sub-Saharan Africa and mass Plio-Pleistocene migration of various African species. Mutation analysis in SIVs present in the South African vervets showed a striking divergence of SIVagm around the Drakensberg Mountain range, suggesting that the mountains represented an insurmountable geographical barrier (due to the lack of water and food resources) during the vervet and SIVagm spread, the timing of which could accordingly be established. The molecular clock based on the phylogenetic relationships among *Chlorocebus* SIVs and further geologically calibrated based on the Bioko split of SIVdrl of the drill monkey (*Mandrillus leucophaeus*) further narrowed down the time of divergence of vervets in South Africa to approximately 200,000 years (6). That allowed time for long-term selective pressure from the SIV pathogen to take place in the African vervets.

Cross-species transmission from NHPs to humans is not a very rare event, as demonstrated by the observation that, during the last century, multiple cross-species transmissions contributed to HIV emergence in humans: two from chimpanzees and two from gorillas, which were responsible for the emergence of the four HIV-1 groups, (M-P) and eight from the sooty mangabeys, which were responsible for the emergence of the eight HIV-2 groups (A-H) (55, 56). In the great apes, SIVcpz emerged following a recombination between SIVrcm from red-capped mangabey (*Cercocebus torquatus torquatus*) and SIVgsn/mus/mon from one of the species on which chimpanzees prey, i.e., greater spot-nosed monkey (*Cercopithecus nictitans*), mustached monkey (*C. cephus*), and mona monkey (*C. mona*), respectively (57). Such chimeric viruses emerge during superinfections/coinfection with different SIVs from different hosts, which enable recombinations (58–60).

Two out of four chimpanzee subspecies in West-Central and East-Central Africa, *Pan troglodytes troglodytes* and *P. t. schweinfurthii*, are infected with SIVcpz (SIVcpz*Ptt* and SIVcpz*Pts*, respectively) at a prevalence of 6%-14%, respectively (60–64), although the distribution of the virus is very uneven between different chimpanzee troops (60). Phylogenetic studies revealed that only SIVcpz*Ptt* is an ancestor of HIV-1 (61, 65). This virus crossed the species barrier to humans in at least two instances, being responsible for the emergence of two HIV-1 groups: M (major), which likely emerged in the first half of the twentieth century (66) and is responsible for the world AIDS pandemic (estimated 98% of HIV infections worldwide) (55); and the non-pandemic HIV1 group N, limited to a small number of infections in Cameroon (61, 67, 68). The HIV-1 groups O and P originated following cross-species transmission of the SIVgor, a virus that naturally infects wild western-lowland gorillas from Cameroon (SIVgor) (69, 70). SIVgor also originated from cross-species transmissions of SIVcpz*Ptt* (69, 71).

HIV-2, comprising eight groups, originated following eight cross-species transmissions of SIVsmm from sooty mangabeys (72–76). Interestingly, SMs naturally infected with SIVsmm were the origin of the major reference strains in macaques (77, 78), following accidental transmissions that occurred during kuru (79) and leprosy (80) experiments carried out in the 1970s in the National Primate Research Centers in the US (81). This accidental experimental transmission of these viruses (which involved serial passages) is probably the reason for their high pathogenicity in macaques (82). Note that direct cross-species transmission of SIVsmm to rhesus macaques may have a very variable pathogenic outcome, ranging from pathogenic infection to virus control (Apetrei, unpublished). Other SIVs are completely controlled when experimentally transmitted to RMs (83–85). Among the different species of macaques, the pigtailed macaques appear to be the most permissive to cross-species infections, as shown by the persistent infections with SIVagm or SIVrcm in PTMs (86–88). The course of progressive SIV infection in macaques recapitulates all aspects of HIV-1 infection in humans, albeit in a more condensed timeframe, and as such, the macaques represent a pathogenic NHP model of simian AIDS (40, 89).

## 3 The clinical course of SIV infection in well-adapted and more recent natural hosts—lessons from the wild

### 3.1 Well adapted hosts, i.e., the vervet/AGM (genus *Chlorocebus*)

AGM, also called the savanna monkey (vervet), is a term that is broadly applied to members of the major subspecies of the single-species genus *Chlorocebus* of the Old World monkey superfamily (90). They are highly abundant primates ranging across sub-Saharan Africa, with the exception of tropical forests and deserts, and heavily infected with species-specific SIVagm (SIVsab in *C. sabaeus*, SIVgri in *C. aethiops*, SIVtan in *C. tantalus*, SIVmal-ZMB in *C. cynosuros*, and SIVver in *C. pygerythrus*) (6, 52, 91). Extensive laboratory data gathered from experimental SIVsab infection in Caribbean AGMs demonstrates that a benign course of the infection characterized by high peak viremia of 10^7^-10^8^ copies/ml 8-10 days postinfection (dpi) that drops to set point values of 2x10^5^ copies/ml by 28 dpi, a transient CD4^+^ T-cell decrease in the blood and lymph nodes, and a lack of systemic T-cell activation (22). In AGMs, inflammatory responses (IL-10 and INF-γ) are activated immediately yet transiently, while lagging in the progressing SIVmac251 infection of the RMs (44). The gut is a major replication site for SIVagm, yet virus is detectable across various tissues, including blood and lymph nodes (the virus from these tissues has more restricted growth in human T-cell lines) and brain and CSF (the virus from these samples is capable of infecting macrophage cultures) (16). However, despite markedly high virus levels in the nervous system, no neuropathologies were observed in AGMs (16).

SIV infection in wild AGM populations, in particular, in South African *C. pygerythrus* (6) and West African *C. sabaeus* in the Gambia (52) has been broadly studied using minimally invasive procedures to assess the markers of chronic immune activation, microbial translocation, abundances of different cell populations, and gut and genital microbiome (6, 52, 92). A combination of viral load measurements and serological testing (ELISA for gp41 peptide) in the plasma allowed the staging of SIV infection (acute/chronic) in these cross-sectional samples based on the Fiebig criteria, according to which chronic infection is diagnosed based on the moderately high viral loads in the presence of anti-SIV antibodies, and acute infection can be defined based on high viral loads in the absence of anti-SIV antibodies (93). The major findings of these pathogenesis studies in the wild (the only studies ever performed in wild animals) were:

#### 3.1.1 Massive yet uneven distribution of SIV infection across demographics

Wild AGMs showed a high prevalence of the SIV infection in adults, with a strong bias towards a higher infection rate in females than males (SIV prevalence in females was 78% in South Africa and 90% in The Gambia, while in the males, the prevalence was significantly lower: 57% in South Africa, and 36% in the Gambia) concordant with unequal male access to females and possibly an increased transmissibility to females (6, 52) [a similar bias to that observed for HIV-1 transmission in humans (94)]. The high frequency of SIV infection was also associated with a very active SIV transmission in the wild populations, as suggested by the high frequency of acute infection (leading to an estimate of SIVver incidence of 4.4% in South Africa) (6).

#### 3.1.2 Exposed seronegative phenomenon

Interestingly, a large proportion of reproductively active adult females (10-22%) avoid SIV infection through heterosexual transmission (6, 52), yet it remains unknown whether this apparent resistance is transient or permanent. It would be interesting to evaluate what factors (e.g., host genetic variants, gene expression, epigenetic modifications, and/or genital and gut microbiomes are associated with the resistance) are protecting the ESN individuals against SIV acquisition and how they are shaping the key features of viral resistance.

#### 3.1.3 Limited mother-to-infant transmission of SIVs in natural hosts in the wild

SIV infection is nearly absent in infants and very rare in juveniles, intermediate in young adults, and high in adults, pointing to a very rare MTIT in the wild and suggesting that the vast majority of transmissions occur around the time when monkeys reach sexual maturity, join the reproductive community, and become exposed to the virus *via* heterosexual contacts and aggressive behavior (6, 52, 95, 96). The very low incidence of SIV MTIT has also been confirmed in captive AGMs, SMs, and MNDs (97–100).

This bias implicates the protection of immature individuals through adaptive biological mechanism(s). CCR5, the main co-receptor of SIV and HIV, shows a striking age-specific expression pattern in CD4^+^ T cells, rising from nearly absent in young individuals to highly abundant in adults (52). Additionally, infected young individuals tend to show higher CCR5 expression in CD4^+^ T cells than uninfected ones (52). The age-related maturation of CCR5 expression in CD4^+^ T cells is associated with a similar age-related increase in the frequency of SIV infection, which points to target cell restriction as a critical defense mechanism against the lentiviral infection of immature individuals (53, 101). This hypothesis was confirmed in experimental mucosal transmission studies in AGMs (51). In experimental studies of breast-feeding transmission of SIVmnd-1 in infant mandrills, low CCR5 expression in CD4^+^ T cells was reported to be the main determinant of the lack of SIVmnd-1 transmission to the offspring (54).

#### 3.1.4 Lack of hallmarks of HIV disease progression to AIDS in the natural environment

SIV infection status (infected/uninfected) in wild AGMs in Africa does not impact the following major health-related measurements (6, 52) (1): body mass index (a proxy for clinical phenotypes not available in field conditions) (2); physiological measures, which are biomarkers of mortality and disease progression in HIV/SIV disease in progressing infection, such as sCD14 (a biomarker of macrophage activation) (102), a wide panel of cytokines and chemokines (103), and C-reactive protein (CRP, a negative biomarker of survival time in HIV-infected patients) (104) (3); the blood counts of major immune cell populations (including CD4^+^ T cells, CD8^+^ T cells, B cells, NK cells, myeloid cells, and plasmacytoid dendritic cells), which do not differ between SIVsab-infected and uninfected AGMs (52). T-cell activation biomarkers (such as the expression of HLA-DR and Ki-67 in CD4^+^ and CD8^+^ T cells) are similarly expressed in SIVsab-infected and uninfected AGMs (52). The three following biomarkers stood out as being associated with SIV infection in African AGMs: D-dimer (a hypercoagulation biomarker), the levels of which are positively correlated with SIV infection, although D-dimer also increases with age and, as such, considering the age-related bias in SIV infection prevalence, it is a reflection of age rather than SIV infection status; IL-6 (an inflammatory cytokine), which is elevated in SIV-infected individuals, but this signal is driven by acute infection, when the high levels of viral replication trigger a transient increase in systemic inflammation in the natural hosts; and CCR5 expression in circulating CD4^+^ T cells; the increase with age of CD4^+^ T cells appears to be a very effective mechanism for preventing MTIT in natural hosts of SIVs (6, 52, 101).

In summary, the absence of differences in the levels of biomarkers associated with disease progression and mortality in natural NHP hosts of SIVs demonstrate that in the presence of other pathogens and parasites, even under harsh natural conditions and sometimes with limited water and food resources, African NHPs that are natural hosts of SIVs show resilience to immunodeficiency upon SIV infection (6, 52).

### 3.2 Vulnerable seminatural hosts—the chimpanzee

SIVcpz infection is pathogenic and manifests with an increased mortality rate (10-16-fold), lower birth rates, and higher infant mortality in natural chimpanzee populations (62, 105). A high rate of SIVcpz infection (40-50%) was associated with local chimpanzee population decline (62). Some SIVcpz-infected chimpanzees in the wild showed lymphatic tissue destruction, depletion of cortical and paracortical T- and B-lymphocytes, and follicular hyperplasia with prominent germinal centers (105, 106). Full-blown AIDS was reported from necropsies of several SIVcpz-infected wild-living chimpanzees that died as a result of AIDS, conspecific (i.e., within species) aggressive behavior, and injuries. Captive chimpanzees showed a blend of features characteristic of benign and pathogenic lentiviral infections. On the one hand, they showed a lack of increased mortality (some individuals were surviving 25 years post infection), no increase in the plasma levels of the sCD14 marker of microbial translocation during chronic infection (yet no direct assessments of SIVcpz showed whether there is indeed a lack of intestinal damage), a lack of AIDS-like clinical signs, and limited immune activation typical of non-progressing infection in well-adapted natural hosts. Yet, captive SIVcpz-infected chimps also displayed reduced CD4^+^ T-cell abundance and disruption of secondary lymphoid tissue architecture typically found in pathogenic infection (107). Soluble markers of immune activation (β-2 microglobulin, neopterin, and sTRAIL) and markers of T-cell activation in the peripheral blood (Patr-DR MHC class II and Ki-67) showed a temporary increase during acute infection, but no lasting increase in the chronic phase, while CD69, also a conventional marker of T-cell activation, showed a fluctuating increase during acute and chronic infection (107). These combinations of pathogenic and non-pathogenic features of lentiviral infection in captive chimpanzees infected with SIVcpz may be associated with advantageous/favorable conditions in controlled environments, i.e., reduced exposure to other pathogens, parasites, conspecific aggression and trauma, availability of food resources (107). In the case of SIVcpz infection of chimpanzees, studies in the wild and a long follow-up decisively contributed to the definition of the pathogenic nature of SIV infection.

The immunopathogenicity of SIVcpz was documented in a western chimpanzee (*Pan troglodytes verus*, a subspecies that does not harbor SIV in the wild) that was experimentally infected with SIVcpz for 20 years. The chimpanzee developed several clinical hallmarks of AIDS (high viremia [10^5^-10^6^ SIVcpz copies/ml of plasma], massive CD4^+^ T-cell depletion [220 cells/ul], and thrombocytopenia [90,000 platelets/ul]), and was effectively treated with antiretroviral therapy (108).

In conclusion, existing data point to the gradual adaptation of the NHP host to their SIVs, i.e., the longer the time of coevolution between SIV and its host, the more effective the host defense mechanisms are at counteracting SIV pathogenic potential and preventing immunodeficiency.

## 4 Entry co-receptor usage

To avoid pathogenic SIV infection, natural hosts have developed adaptations that control the use of the CCR5-mediated entry pathway (101).

### 4.1 CCR5 genetic deficiency and co-receptor use

Natural hosts have acquired CCR5 null mutations that, similar to the CCR5-Δ32 loss-of-function allele in human populations that can modulate the risk of HIV transmission (109–111), abrogate CCR5 surface expression and protect null homozygotes from SIV entry through the CCR5 pathway [CCR5-Δ24 with a frequency of 86.6% in RCMs, 4.1% in SMs (112) and CCR5- Δ2 in SMs (113)]. Yet, the SIV pathogen bypasses the lack of functional CCR5 co-receptor in some RCMs and SMs and utilizes the non-CCR5-mediated entry pathway with alternative co-receptors. Thus, SIVrcm in RCMs uses CCR2b as the main entry co-receptor, while SIVsmm in SM can use CXCR6 as an alternative co-receptor in addition to CCR5 (113).

As a result of CCR5 downregulation, SMs and AGMs have significantly lower levels of CD4^+^ CCR5^+^ T cells than humans or RMs, both in the blood and at mucosal sites (53). The shift in tropism from CCR5 to CXCR6 may benefit the host, as it directs the virus to a different and putatively more dispensable cell population (113). On the other hand, the loss of CXCR6 co-receptor use is characteristic of the lineage of more pathogenic lentiviruses—SIVcpz in chimpanzees and HIV-1 in humans (114).

### 4.2 Restriction of infection through control of surface expression of canonical receptors

Restricted expression of entry receptors eliciting resistance to SIV infection is a mechanism that helps to spare critically important cell populations among the CD4^+^ T lymphocyte memory pool—the long-lived central memory CD4^+^ T cells (Tcm). Tcm T cells have stem-like properties, reside primarily in the lymphoid tissue, and have strong proliferation capabilities in response to stimuli. In stark contrast, effector memory CD4^+^ T cells (Tem) have lower proliferation potential, are located in non-lymphoid tissues, and remain susceptible to SIV infection (5).

In SMs, CD4^+^ Tcm cells are relatively protected from SIV-mediated depletion through the downregulation of CCR5 (115). CCR5, which is the canonical co-receptor for HIV/SIV infection in humans and RMs, is also expressed in the CD4^+^ T cell of the natural hosts (SMs, AGMs, mandrills, and sun-tailed monkeys) but at very low levels (53). Instead of CCR5, CXCR6 is the major SIV co-receptor in SMs and AGMs (116–118). As a result, SIV still can infect and replicate in natural hosts but only in the cell populations expressing CXCR6. The targeting of different cell populations by SIV is dependent on the pattern of distribution of the co-receptors CCR5 and CXCR6. In SMs, they are expressed in largely non-overlapping populations of CD4^+^ T cells, with only 0.3% of CD4^+^ T cells being dual-positive for CCR5 and CXCR6 (114). In CD4^+^ T cell memory subsets, CXCR6 was enriched in Tem (5.9%) but less so in naive CD4^+^ T cells (0.5%) and Tcm (1%) (114). Interestingly, some features of non-progressing SIVsmm infection in SMs—low immune activation despite high viremia and low CCR5 density on CD4^+^ Tcm cells—were observed in a pediatric HIV non-progressor cohort from South Africa (119). The fine-tuned control of co-receptor expression helps to preserve the CD4^+^ Tcm population, which is critical for maintaining immune homeostasis. A similar phenomenon of protecting Tcm CD4^+^ cells from SIV infection is achieved through the downregulation of CD4 in CD4^+^ Tcm cells in AGMs (48, 120).

## 5 Preservation of gut health in SIV-infected NHPs that are natural hosts

### 5.1 Different impacts of SIV infection on the intestinal mucosa between natural and non-natural hosts

Salient features of non-pathogenic SIV infections in natural hosts that differentiate them from pathologic infections include preservation of the mucosal barrier, preservation of the Th-17 cells (the T-cell subset that protects mucosa against bacterial and fungal infection) in the gut and periphery (31, 32), a lack of microbial translocation, and a lack of chronic inflammation and T-cell immune activation (121–123), which suggest that gut health is a key determinant of the pathogenic or non-pathogenic course of infection.

#### 5.1.1 Integrity of the Mucosal Barrier

##### 5.1.1.1 The role of microbial translocation

The gut plays a dual role as a structural and immunological barrier between the external and internal environment. It requires a fine-tuned balance between tolerance and sensitivity and the ability to provide rapid yet sustainable long-term protection against pathogens (124, 125). Nearly 70% of the body’s T-cell-generating lymphoid tissue is located in the gut (124), which is also the primary site of SIV/HIV replication (25, 43, 126–128), and intestinal epithelium is highly susceptible to inflammation. Lentiviral infection leads to physical and immunological dysfunction of the intestinal mucosa (129, 130). Damage to the enteric barrier allows microbial and fungal translocation, i.e., the leakage of microbial and fungal products across the breached intestinal epithelium into the blood circulation, and causes the systemic dissemination of microbial biomolecules, such as LPS (bacterial translocation marker), peptidoglycan, bacterial DNA, and viral genomes, which can be found in various tissues even at distant anatomical sites with progressive SIV infection (131) and, in the case of the major fungal cell wall antigen β-d-glucan (BDG) (a biomarker of fungal translocation), at elevated levels in the plasma (132, 133).

Microbial products translocated from the gut strongly stimulate the immune system and the production of cytokines, contributing to persistent local and systemic inflammation and T-cell activation, which further amplify viremia by generating more target cells in both treated and untreated HIV-infected individuals (43). While initially CD4^+^ T-cell counts or plasma viral loads appeared to be the most accurate biomarkers of disease progression, immune activation that enhances virus replication by providing the virus with activated CD4^+^ T-cell targets is now considered a more accurate predictor of survival time in advanced HIV-1 disease (134). Note that persistent CD4^+^ T cell depletion for over 1.5 years did not result in SIV disease progression in AGMs (135). Plasma levels of soluble CD14 (a biomarker of microbial translocation) are an independent predictor of mortality in HIV infection (102). Microbial translocation is characteristic of the disease pathogenesis of not only acquired immunodeficiency (HIV infection) (136) but also primary deficiencies, such as idiopathic CD4^+^ T-cell lymphocytopenia (ICL) (137); however, the link between microbial translocation and the perturbation of CD4^+^ T cell homeostasis in ICL is not as obvious as in the case of HIV-1/AIDS. In ICL, sCD14 levels are only modestly increased, and the local architecture of the GI tract is preserved, with normal enterocyte turnover and no apparent loss of Th17 cells, which is in contrast to patients with HIV-1/AIDS (138).

BDG is a biomarker of fungal translocation and is elevated in HIV-infected individuals (139) and is associated with gut damage, immune activation and inflammation (140), and a risk of neurocognitive comorbidities (141). In HIV-infected individuals, fungal products stimulate antigen-presenting cells (monocytes and macrophages) and NK cells, leading to the excessive secretion of proinflammatory cytokines and inflammation (133).

The following mechanisms may be responsible for triggering the functional loss of the gut barrier in progressive HIV/SIV infections (142) (1): loss of the CD4+ T cells, the primary target of HIV-1/SIV infection, undergoing virus-induced cell death, which results in a severe reduction of this cell population in circulation and mucosal sites during acute infection in both progressive and non-progressive hosts (22, 23, 27, 53, 143) (2); preferential infection and loss of Th-17 cells, which play a critical role in protecting against microbial and fungal pathogens and regenerating the gut epithelium, thus being a key contributor to the maintenance of mucosal integrity (144) in the GI tract. Differentiation of Th-17 cells and HIV replication in these cells are regulated by a retinoic acid-related nuclear hormone receptor, RORC2 (145). The long-term survival and proliferation capacities of Th-17 cells make them preferential targets for HIV reservoirs in individuals receiving ART (146, 147). By contrast to progressing infection, Th-17 abundance is well maintained in the natural hosts during non-progressing infection (31, 32) (3); persistent gut cell death and mucosal apoptosis (130, 148, 149). The loss of integrity of the intestinal immune barrier occurs during the acute pathogenic infection and continues through the chronic phase due to incomplete repair of the barrier. These gut breaches permit an influx of microbial toxins into the circulation, which drive chronic T-cell immune activation and systemic inflammation. In contrast to the progressing hosts, natural hosts chronically infected with species-specific SIVs (SIVsmm-infected SMs and SIVagm-infected AGMs) manage to prevent permeabilization of the mucosal barrier (23, 28). As such, gut integrity is maintained in the natural hosts throughout the SIV infection (150, 151). As a result, in spite of the relatively robust viral replication resulting in levels of viremia, which are similar or higher than those observed during pathogenic SIV infection, the natural hosts of SIVs do not display any of the hallmarks of HIV disease progression, i.e., breakdown of the gut epithelial barrier, microbial translocation, and chronic T-cell immune activation and inflammation, CD4^+^ T-cell decline, or hypercoagulation (6, 28, 52, 122, 130, 151–153).

**Figure 2:**
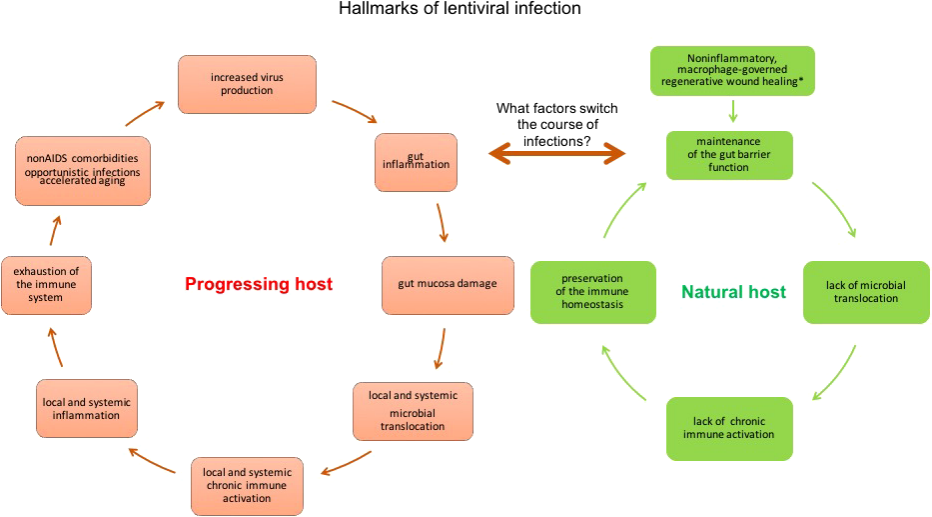
Hallmarks of lentiviral infection in natural non-progressing hosts (vervets/AGMs and SMs) and progressing hosts (humans and RMs). In the acute phase of infection in vervet/AGMs, the immediate activation of macrophage-associated regenerative wound healing bypassing the early inflammatory stage prevents the loss of immune barrier function of the gut (156). However, chemically induced damage of the intestinal mucosa with DSS treatment during acute infection shifts the course of pathogenesis from a non-pathogenic course (controlling microbial translocation and its consequences of persistent activation of the immune system) to a pathogenic course (losing the containment of the luminal microbiota, leading to immune activation and inflammation on a local and systemic scale), demonstrating that dysfunction of the gut barrier can initiate a vicious cycle in SIV-infected non-progressing hosts (157). Whether the DSS-induced hallmarks of the pathogenic course of infection in AGMs are predictive of the development of HIV-like clinical manifestations typical to progressing infection, needs to be determined. Longer DSS administration is needed to determine whether that would be sufficient to evoke progression to AIDS.

Alterations of the mucosal barrier of SIV-infected natural hosts drive the otherwise non-pathogenic infection towards disease progression. The intestinal mucosa functions as a physical and immunological barrier and is thus critical for gut and systemic health. All impairments of gut integrity, such as pathogenic lentiviral infections, inflammatory bowel disease (IBD) (154), are associated with chronic systemic inflammation and immune activation (124, 125). Damage to the intestinal mucosa and loss of its barrier function can occur in the absence of lentiviral infection during multiple clinical conditions, including celiac disease, IBD, colon carcinoma, chronic liver disease, type 1 diabetes, and obesity (155) (**Figure 2**). Experimental destruction of the mucosal barrier of uninfected RMs with dextran sulfate sodium (DSS), which is toxic to enterocytes in the crypts and causes chemically induced colitis in rodents and non-human primates (157), induces gut damage that evokes features of pathogenic lentiviral infection, including neutrophil infiltration of the lamina propria in the colon, microbial translocation to local and distant anatomical sites, local inflammation and activation of local T cells, systemic inflammation (IL-6) and immune activation, and fibrosis in lymphoid tissues, which is a histopathologic hallmark of pathogenic lentiviral infection (157).

Furthermore, the administration of DSS to chronically SIVagm-infected AGMs induces a gut dysfunction (mucosal thickening, redness, ulcerations, and colitis) that can drive the non-pathogenic SIV infection towards a more progressive pattern. This experimentally induced gut damage is associated with elevated levels of the plasma markers of gut barrier dysfunction, microbial translocation, and systemic immune activation (sCD14) and inflammation (CRP) that further increases plasma viral loads and CD4^+^ T-cell depletion (157). Note, however, that while chemical-induced gut dysfunction clearly has the potential to alter the clinical pattern of SIV infection in natural hosts, studies of longer DSS administration are needed to test whether or not the induced defects of the mucosal barrier are sufficient to trigger progression to AIDS.

##### 5.1.1.2 Robustness of the intestinal barrier

Intestinal barrier robustness was tested in a single study that assessed, on serial necropsies, systemic health during the acute phase of non-pathogenic SIVagm infection of AGMs (151). The observations derived from this study were as follows: (1) SIV infection exerted a minimal impact on the major immune populations both in the periphery and at the mucosal sites. Thus, circulating CD4^+^ T cells were only minimally depleted during acute SIVagm infection, and were already restored to pre-infection levels by the time of transition to chronic infection; (2) very importantly, and in agreement with previous observations (158), CD4^+^ T-cell loss through bystander mechanisms was not increased in SIV-infected AGMs. Thus, little or no apoptosis in the gut lamina propria and epithelium was observed (based on the caspase-3 biomarker). This minimal loss, in the absence of bystander depletion mechanisms, did not induce a major local inflammation; instead, (3) local inflammation of the gut mucosa was very transient, and likely virus induced, as demonstrated by a temporary increase of MPO-neutrophils that returned to the baseline after the peak of viremia, an increase of MX-1 at the peak of viremia, and a transient increase of Ki-67 expression in the lymph nodes at the peak of viremia during the chronic phase; as a result, (4) the overall integrity of the mucosal barrier was maintained, as demonstrated by the overall preservation of the continuity of colonic epithelium, which was documented by the distribution of claudin-3, a biomarker of the proper sealing of tight junctions, which is critical for regulating the permeability of the epithelial barrier; (5) microbial translocation did not increase with SIV infection, as indicated by biomarker analysis (based on *in situ* LPS and an *E. coli* IHS assay), or at best was transiently increased around the peak of virus replication (based on the plasma levels of LPS and sCD14). (6) the absence of SIV-induced fibrosis in the gut or lymph nodes was documented by the lack of increased collagen deposits at these sites and normal plasma levels of hyaluronic acid (a biomarker of liver fibrosis) (151). These findings underscore that the key distinction of the natural host’s response to the lentiviral infection is the capability to withstand SIV infection without damage to gut mucosa integrity, rather than rapidly repair and restore breaks in it (151). This might be because of superior healing mechanisms that enable AGMs to sustain gut integrity without permanent or even transient damage (156).

##### 5.1.1.3 How do the natural hosts prevent gut dysfunction?

The issue of how natural hosts prevent gut dysfunction was investigated through comparative transcriptomic analysis of responses to SIVsab and SIVmac in the rectal tissues of AGMs and RMs, respectively, during acute infection (156). Gene expression responses characteristic of the pathogenic infection of RMs were primarily orchestrated by LPS as the main upstream regulator and showed a stronger activation of antiviral and antimicrobial immune responses. By contrast, reactions specific to the non-pathogenic infection in AGMs did not manifest antimicrobial signatures, but appeared to be driven by IFN-γ and prolactin genes (both inflammatory cytokines) as the upstream regulators and were marked by the recruitment of leukocytes and myeloid cells. AGMs activated IFN-driven targets, myeloid cell migration, and the developmental processes of muscle, epithelial, and adipose tissues in the absence of microbe-activated patterns as early as the previremic phase, suggesting that preventive repair mechanisms may be responsible for stopping microbial translocation (156).

The superior ability to maintain the gut barrier function of the gut mucosa throughout acute infection is a critical feature that differentiates pathogenic and non-pathogenic infection (151). Transcriptomic profiles in rectal tissues through the course of non-pathogenic SIVsab infection in AGMs pointed to regenerative wound healing mechanisms, which bypass the early inflammatory processes and rapidly activate the late stage of wound healing, resembling the transcriptional profile that regulates the regeneration process in the aquatic axolotl, with fully functional scar-free repairs, including blood vessel development (angiogenesis). This process in AGMs involved fibronectin as a key connector between collagen and the laminin components of the extracellular matrix in transcriptional networks. Among the predicted upstream regulators of the wound healing networks was EGFB release factor, which was also upregulated during the axolotl wound repair process, suggesting that TGF-β secretion is a regulator of monocyte/macrophage-mediated epithelial cell repair in AGMs (156).

Taken together, while pathogenic infections are characterized by inflammatory tissue damage signatures and microbial pattern recognition receptor and inflammatory signaling, non-pathogenic hosts displayed a strong wound-healing signature, probably regulated by monocytes early in the acute infection. This wound-healing ability prevented infection from taking a pathogenic course and causing microbial translocation and subsequent local and systemic inflammation and chronic immune activation (156).

#### 5.1.2 The microbiome

The development and health of the immune system is shaped by commensal microbiome, and disruption of its homeostasis leads to the impairment of the immune system. The intestinal ecosystem is the site of multidirectional interactions between the host, the commensal microbiome, and the pathogen. Progressive loss of gut immune function (due to loss of CD4^+^ T cells in the gut mucosa, in particular the loss of the Th-17 CD4^+^ T cells that are key effectors that maintain immune barrier function and memory cells) contributes to the destabilization of the intestinal microbiome in HIV-infected individuals (159). A shift towards dysbiotic mucosa-associated communities enriched in *Proteobacteria* and depletion of *Bacteroidia* species was associated with mucosal immune disruption, T-cell activation, and chronic inflammation in HIV-infected subjects (160). Biomarkers of HIV disease progression, such as levels of mucosal and systemic immune activation and inflammation, CD4^+^ T-cell counts, and viral loads, were correlated with gut dysbiosis manifesting with the disruption of microbial homeostasis, characterized by an expansion of pathogenic bacteria compared with commensal species (160–162). However, various studies produced diverse results, probably due to variability within and between the study cohorts. Pathogenic infection in RMs led to shifts in the composition of gut microbiota, yet studies lacked consistency, probably due to cohort and experimental variability (163). Nevertheless, the gut microbiome can act as a potential modulator of SIV/HIV disease, probably as one of the main determinants of the resilience to lentiviral-induced destruction of the gut mucosa and microbial translocation and their consequences.

**Figure 3:**
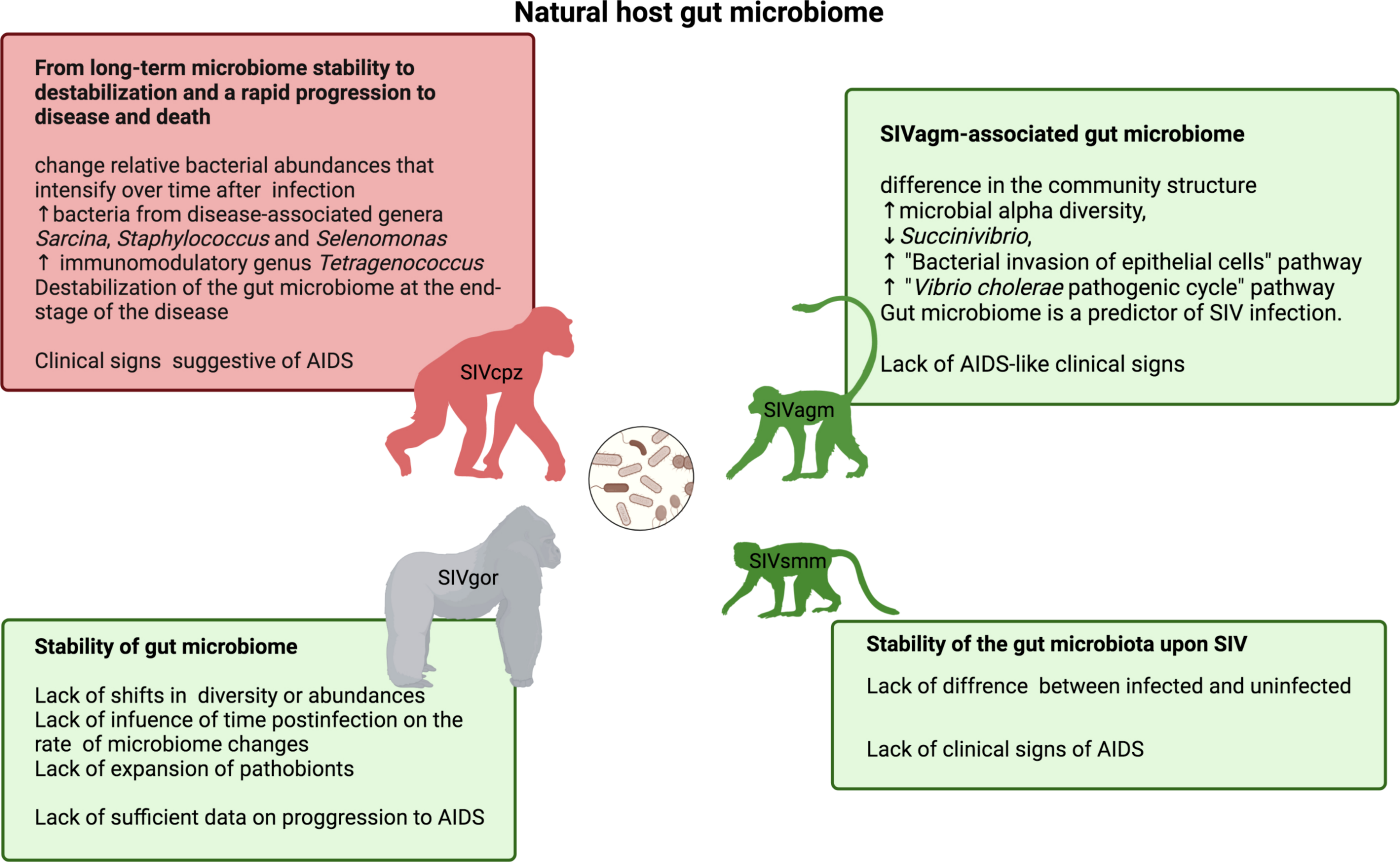
The gut microbiome of natural NHP hosts of SIV infection: chimpanzee, a host displaying mixed clinical signs of progressing and non-progressing SIV infection (top left) (164, 165); gorilla, a host with lacking sufficient data on clinical indicators of AIDS (bottom left) (166); and hosts that typically do not develop immunodeficiency and simian AIDS - vervet/AGM (top right) (92) and sooty mangabey (bottom right) (167).

The extent to which the gut microbiome helps natural hosts preserve the integrity of the gut mucosa and prevent translocation of bacterial products from the gut is not fully understood, but there is increasing insight into the natural microbiome composition in wild populations of natural hosts (**Figure 3**). We anticipated microbiota stability or activation of protective mechanisms in ancient well-adapted hosts (SMs and AGMs) and destabilization of the microbiome in more recent hosts (CPZ).

##### 5.1.2.1 The impact of SIVcpz on the chimpanzee gut microbiome

SIVcpz impact on the gut microbiomes of chimpanzees was studied in wild eastern chimpanzees (*Pan troglodytes schweinfurthii*) from Gombe National Park, Tanzania that naturally host pathogenic SIVcpz*Pts* (164). Long-term non-invasive monitoring of SIV infection status and AIDS-like symptoms in this chimpanzee population allowed the assessment of changes in the fecal microbiome upon SIVcpz*Pts* infection (164). While the core gut microbiome suggested a composition that was overall stable at the phylum level, with no significant changes in the abundance of individual bacteria upon SIVcpz*Pts* infection, it underwent marked changes in relative microbial abundance that increased with time and an upturn in microbes not observed prior to infection and potentially relevant to immune health, i.e., bacteria from disease-associated genera (*Sarcina*, *Staphylococcus* and *Selenomonas*) and *Tetragenococcus* (164), known for their immunomodulatory effect (168). Further studies using a combination of metagenomic sequencing and larger-scale bacterial 16SrRNA gene sequencing showed that the chimpanzee gut microbiome is very robust throughout most of the course of SIV infection and that its stability collapses only in the final stage of disease and for the few months preceding AIDS-related death (165). SIVcpz-infected chimpanzees showed a tendency towards enrichment for bacteria from the *Prevotellaceae* family (165). Chimpanzee stool-associated circular virus (Chi-SCV) and adenovirus (ChAdV), quantified using metagenomic sequencing, did not show differential abundances in SIVcpz-infected chimpanzees (165). The microbial composition in the guts of SIVcpz-infected chimpanzees showed continuous drifts, resulting in gradual changes in response to SIVcpz infection in wild chimpanzees rather than a single shift.

##### 5.1.2.2 Stability of the gorilla gut microbiome

Non-invasive sampling methods investigating SIV infection status and evaluating SIV pathogenicity *via* microbiome analysis in feces provided insight into the influence of SIVgor infection in the gut microbiome of western lowland gorillas (*Gorilla gorilla gorilla*). While SIVcpz in chimpanzees and HIV-1 in humans are pathogenic and cause substantial alterations to the composition of the gut microbiome (164, 165), it remains unknown whether the closely related SIVgor leads to pathogenesis and clinical signs of AIDS in its natural host. Despite SIVgor originating from the SIVcpz strain, which destabilizes the gut microbiome in chimpanzees, SIVgor infection in gorillas does not show similarly pathogenic behavior and does not lead to gut microbiome variation in western gorillas from Southern Cameroon. Unlike chimpanzees, the gorilla gut microbiome does not show a progressing shift in composition over time or a tendency towards the emergence of opportunistic pathogens upon infection (166). There were no noticeable differences in diversity and composition between SIVgor-infected and uninfected gorillas or accelerated changes in microbiome composition over time in infected individuals.

##### 5.1.2.3 SIVver-associated variation in the microbiomes of South African AGMs

Typically, 5-7 taxa are delineated within the genus *Chlorocebus*, which are characterized by different geographical ranges across tropical and temperate climatic zones in Africa and a massive spread of SIVagm infection (90). However, the majority of controlled experiments are conducted in AGMs originating from a founder isolate population in the Caribbean Islands (St. Kitts and Nevis) that was more recently established from the west African populations and is free of SIV (169, 170). So far, microbiome studies relating to SIV infection have been conducted only in the natural populations of South African AGMs (*Chlorocebus pygerythrus*) (92). Studies of natural microbiome in wild vervets involved a wide range of ages, from infants to old adults, to cover as much as possible the epidemiological profile of SIV infection/transmission and the course of the diseases (6). While SIV-infected vervets (both in natural and captive populations) do not experience gut dysfunction, microbial translocation, and chronic immune activation and in general do not progress to immunodeficiency, their microbiome displays significant differences in the microbial ecosystems of the gut and genital tract compared with uninfected individuals (92). The gut microbiota in SIV-infected vervets showed (1) increased alpha diversity (2), decreased abundance of the phylum *Proteobacteria* (particularly the genus *Succinivibrio*) (3), decreased ‘bacterial invasion of epithelial cells’ and ‘*Vibrio cholerae* pathogenic cycle’ KEGG pathways in the predicted fecal metagenome of SIV-infected individuals compared with uninfected individuals, and (4) partial control of early SIV-induced alterations during chronic infection (92). The host microbiome differences between SIV-infected and uninfected individuals may represent adaptations to the virus that prevent microbial translocation, persistent immune activation, and the resultant disease progression in vervets. This cross-sectional study showed that SIVver infection in vervets is characterized by a distinct gut microbiome composition and functionality yet did not determine whether these differences are pre-existing or responsive to the infection, a question that could be further addressed through longitudinal studies.

Fecal samples were an accurate predictor of SIV infection status in wild vervets (92). While this observation was obtained from samples collected from the rectum, it seems possible that non-invasively collected samples on the ground may provide a feasible microbial biomarker predictive of immune health in wild primate populations.

Conventional studies of disease correlates in animal models, including AGMs, are typically conducted under well-controlled standardized laboratory conditions in a small number of animals that can be subjected to invasive sampling. Natural populations, on the other hand, allow for more scalable studies (dozens or even hundreds of individuals) in the context of a complex and variable environment (including natural habitat and social groups) using non- or minimally invasive sampling. The natural setting differs from the laboratory as wild primates usually expend more energy and may ingest less nutritional food, which is highly dependent on timing, habitat, geographic location, and seasonality of environment, and aggressive interactions in the group may reduce equal access to foraging. In wild AGMs from South Africa, gut and vaginal microbiome revealed an association with geographic location and environmental variables, such as geographical biomes and temperature (92). The within-genus differences may be significant, as illustrated by differential/distinctive vaginal microbiome composition between South African and Caribbean AGM populations (92). Comparison of the effects of diet between Caribbean-origin AGMs in the wild consuming a natural diet and AGMs fed a typical western diet (TWD) under controlled conditions showed similar microbial richness, yet the composition of their microbiome was distinct, with lower abundances of Firmicutes, Lentisphaerae, Proteobacteria, Tenericutes, and Verrucomicrobia and higher abundances of Bacteroidetes and TM7 in AGMs with a TWD (171). Yet, wild populations more closely reproduce conditions reflecting host-pathogen coevolution and are the preferred setting for studies of natural transmission.

##### 5.1.2.4 SIVsmm-infected and -regulated SM gut microbiome

The gut microbiomes of captive SMs did not show significant SIVsmm-associated variation in microbial diversity or community structure, yet there were lower levels of pathobiont bacteria in SMs not infected with SIVsmm than in diet-matched RMs experimentally pathogenically infected with SIVmac239 (in the early chronic phase) (167). The stability of gastrointestinal microbiota was paralleled with the compositional stability of the cargo of the luminal intestinal microvesicles produced by the host enterocytes in chronic SIVsmm infection in SMs, which was in contrast to RMs infected with closely related SIVmac (172). Progressive SIVmac infection in RMs showed shifts in microbial communities concomitant with changes in the content of gut microvesicles compared with uninfected RMs, including a reduced amount of several miRNAs and an increased level of beta-defensin 1 (DEFB1). The microvesicles from the progressive infection hampered the growth of *Lactobacillus salivarius*, one of the commensal bacteria undergoing microbial translocation, suggesting that their potential role is to control microbial growth and shape microbial translocation (172).

## 6 Homeostatic regulation of immune cell populations

A series of phenotypic adaptations, which are considered to have occurred during the millenary virus-host coevolution as tools of the host adaptation to circumvent the deleterious effects of SIV infection, characterize the immune system of the natural hosts of SIV:

### 6.1 Multifunctional CD4^neg^ T cells

Some natural hosts of SIV (AGMs, SMs, and patas monkeys) harbor an evolutionarily conserved downregulation of the CD4 molecule on the T-cell surface, resulting in low levels of this cell subset in contrast to its abundance in non-natural hosts of SIV/HIV (47, 48). *Chlorocebus pygerythrus* has large CD4^neg^ CD8αα+ T-cell populations (lacking CD4, and thus spared from SIV infection, and distinct from canonical CD8^+^ T cells expressing heterodimers with α and β chains) (50). Meanwhile, SMs have abundant populations of double-negative cells (expressing neither CD4 nor CD8) that are critical for preventing massive homeostatic alterations during acute CD4^+^ T-cell loss (49, 173, 174). These CD4^neg^ cell populations are virus-resistant and preserve some CD4^+^ T-cell functions (49, 50, 175). In *C. pygerythrus*, the reduced CD4 expression in CD4^+^ T cells may be related to the regulation of RUNX3 expression, the master transcription factor regulating CD4/CD8 expression (175). Runx3 is involved in CD4 suppression in murine models; in RMs, its expression is higher in CD8αβ T cells than in CD4^+^ T cells. Yet, in vervets, RUNX3 expression does not vary among all T cell subsets (including, CD8αα^+^ T cells, CD4^+^, and CD8αβ^+^ T cells), irrespective of their CD4 and CD8 phenotype, suggesting that RUNX3 may be the factor contributing to CD4 downregulation in CD4^+^ T cells (175).

The CD4^neg^ CD8αα^+^ T-cell populations of *C. pygerythrus* also display functions characteristic to classical CD4^+^ T cells (MHC class II restriction, expression of FoxP3, CD40 ligand, and production of IL-17 and/or IL-2) (120). These cells can avoid SIV infection while performing some CD4^+^ T cell-like functions in the absence or with very low levels of classical CD4^+^ T-cells. The conversion process is accelerated by SIV infection in AGMs *in vitro* and induced both *in vitro* and *in vivo* by homeostatic cytokines, such as IL-2 (50, 176). These virus-resistant hyperfunctional T cells arise through post-thymical downregulation of CD4 by CD4^+^ T cells causing CD4-to-CD8αα conversion (120). This is a developmental process resulting in the increase of the initially low abundance of CD4^neg^ CD8αα^+^ T cells with age. CD4 downregulation is evoked through epigenetic silencing mechanisms involving CpG methylation in the CD4 promoter (177), which colocalizes with inaccessible chromatin regions in the CD4 gene region, as determined using ATAC-seq (178). Differential chromatin accessibility across the genome attributable to the CD4-to-CD8αα conversion was associated with immune processes and T-cell biology, and binding sites for transcription factors involved in the immune processes. In CD4^+^ memory T-cells, more accessible regions were enriched for high mobility group (HMG) family binding sites involved in T-cell development and differentiation. In CD8aa^+^ T cells, open chromatin regions were enriched for binding sites for Runt domain transcription factor and erythroblast transformation specific (ETS) regulating hematopoiesis (178).

### 6.2 Natural killer cells

Lymph node follicles of the natural host species remain virus-free during SIV infection, in contrast to HIV-infected individuals and SIV-infected NHP progressors, in which they are major viral reservoirs, even in patients on ART (179). This observation raises the following question: what are the protecting mechanisms preventing infection of the cells in the lymph nodes in AIDS-resistant NHPs? The frequencies and phenotypes of NK cells (defined as CD45^+^ CD3^neg^ CD14^neg^ CD20^neg^ NKG2a/c^+^) assessed in African and Asian NHPs were similarly anatomically distributed in NHPs with different origins, yet the frequencies of circulating NKG2a/c^low^ NK cells was higher in African species than in the Asian ones (180). It was also reported that SIV infection differentially drives NK distribution in natural and non-natural hosts: in AGMs, contrary to cynomolgus macaque, NK cells did not home to the gut, and their abundance at mucosal sites was lower during SIV infection but increased in lymphoid tissues (180). This was consistent with an effective control of viral replication in the T zone and B cell follicles in secondary lymph nodes mediated by NK cells occurring in AGMs, in contrast to progressing SIV infection in macaques in which these anatomical locations are the major viral reservoir (181). The process of effective control of SIVagm in the lymph nodes of natural hosts involves SIVagm-induced expansion of terminally differentiated NKG2a^low^ NK cells in lymphoid tissues. These expanded cell subsets showed upregulation of genes in the pathways related to lytic activities of NK cells and displayed adaptive transcriptional profiles (based on marker analysis) and increased MHC-E-restricted cytotoxicity towards cells presenting SIV peptides (182). MHC-E-restricted NK cell activity against target cells presenting SIV peptides was enhanced during SIVagm infection in AGMs but decreased with SIV infection in macaques (182).

### 6.3 NKG2a/c^+^ CD8^+^ T cells

Non-pathogenic SIVagm infection in AGMs is characterized by a rapid expansion of NKG2a/c^+^ CD8^+^ T cells in circulation and at mucosal sites but not in secondary lymphoid tissues (183). The transcriptomic signatures of these expanded NKG2a/c^+^ CD8^+^ T cells are distinctive from other CD8^+^ T cells, implying a cytotoxic effect and immunoregulatory function, as well gut homing properties of these cells. Such a pattern of cellular dynamics was not observed during the pathogenic SIV infection of RMs. In the progressing model, NKG2a/c^+^ CD8^+^ T cell abundancy negatively correlated with the mucosal levels of IL-23, an inflammatory cytokine. This negative correlation between NKG2a/c^+^ CD8^+^ T cells with intestinal inflammation in the pathogenic models suggests that these cells protect against intestinal inflammation during SIV infection by controlling immunopathology in the intestine (183).

## 7 Genetic signatures and pathways associated with SIV infection in natural hosts

What genes and pathways underlie a non-progressive course of SIV infection in natural hosts despite chronic virus replication? Genomic analysis enabled an unbiased genome-wide search for host adaptive links against disease progression in SIV-infected NHPs. Genomic studies revealed genetic adaptations in the genes involved in biological processes acting in response to viral infections in AGMs and SMs.

### 7.1 Genetic adaptations to SIV infection

Comprehensive omics approaches aiming for a complete perspective, such as genome-, transcriptome-, and epigenome-wide studies, facilitate an unbiased identification of the host molecular reactions to infection and adaptive responses against the progression to disease. The identification of molecular processes and key genes driving the ability to evade immunodeficiency may set new directions for the development of novel therapeutic approaches.

Genome-wide studies using next generation sequencing revealed the genetic adaptations in the genes involved in biological processes responding to viral infection, including SIVs, in AGMs (184) and SMs (185–188). Genome sequencing of representatives of diverse vervet populations across major *Chlorocebus* species revealed massive polygenic adaptations to SIV in vervets (6, 184). Genes with strong selection signals were enriched in the GO category ‘viral processes’, vervet orthologs of human genes interacting with HIV, and genes transcriptionally regulated in response to SIV infection in the natural host (AGMs), but not non-natural hosts (RMs). Early expressional responses were enriched for clathrin-mediated endocytosis (a process utilized by HIV to enter cells lacking CD4), autophagosome assembly (autophagy being a key component of host responses to HIV-1 infection), and the regulation of type I IFN production (a key driver of early transcriptomic responses both in natural and non-natural hosts), while expressional changes in the chronic phase were, among others, enriched for the regulation of NK cell activation (184).

Comparative genomic studies contrasting the protein coding regions of SMs with humans and RMs revealed the genes that were most diverged at the amino acid level and in structures between the natural host and pathogenic hosts—intercellular adhesion molecule 2 (ICAM-2) and toll-like receptor-4 (TLR4) (188). Functional studies of the top immunoregulatory proteins displaying major structural differences specific to the natural host demonstrated that sequence differences have significant functional consequences: abrogation of the cell surface expression of ICAM-2 and TLR4 blunted the production of pro-inflammatory cytokines in response to LPS stimulation *in vitro*, a potential factor contributing to reduced immune activation (188). Genomics combined with functional assays allowed the identification of candidate modifier genes shaping the resistance of the natural host to immunodeficiency.

### 7.2 Transcriptomic mechanisms

The benign phenotype of SIV infection in SMs has been associated with genes in pathways contributing to interferon signaling, BRCA1/DNA damage response, PKR/INF induction, and CGALS8 in SMs (189) and point to the leading role of the genes LGALS8 and IL-17RA, which enhance gut barrier function and shape homeostasis with gut microbiota.

Wild NHPs are important models for understanding the host mechanisms involved in the control or pathogenesis of viral infections. Ugandan red colobus (URC) in Kibale National Park are naturally infected with SIVkrc (22.3%) (190). RNA-seq in URC from this wild population pointed to pathways associated with cellular immunity, cell activation, leukocyte activation in infected URC, and inflammatory responses in uninfected URC. SIVkrc infection has been characterized by downregulation of SLAMF6 and CD4, immune genes undergoing downregulation in HIV infection, and upregulation of the immunosuppressive gene CD101 during infection (191). The pattern of temporary upregulation of ISGs observed in AGMs and SMs was not displayed by SIVkrc-infected URC, most likely because in a moderately sized group a vast majority, or all, are in the chronic phase of infection when ISG expression has already normalized (191).

### 7.3 Epigenetic mechanisms

SIV infection has a different influence on DNA methylation patterns in CD4^+^ T cells purified from circulation and lymphoid tissue in non-pathogenic SIVagm infection of AGMs and pathogenic SIV infection of Chinese RMs (192). Non-pathogenic SIV infection of AGMs is characterized by the enrichment for methylation signals in regulatory proteins, while pathogenic infection in macaques is associated with Th1-signaling and metabolic pathways, suggesting that epigenetic mechanism may contribute to the risk of metabolic diseases during progressing infection (192).

## 8 Conclusions and future directions: implications for the development of novel therapeutics

As shown above, studies of natural hosts that remain disease-free despite life-long SIV infection identified several directions towards the development of therapeutic approaches: (1) Ameliorating gut inflammation and damage that leads to aberrant mucosal permeability and translocation of enteric bacteria to the circulation appears to be a promising therapeutic direction. Commensal enteric microbes play an important role in intestinal wound healing and the maintenance of barrier function (193) and can decrease gut permeability associated with low-grade inflammation and normalize the levels of tight junction proteins, such as claudin, in mice (194). (2) Therefore, stabilization of the natural microbiome, preserving and/or enriching beneficial taxa, manipulation of community composition, and metabolic activity is another possible therapeutic direction. Attempts to reduce gut inflammation through modification of the enteric microbiome using selected bacterial strains as probiotics alone or combined with prebiotics, or bacterial metabolic products, such as short-chain fatty acids, brought mixed results (123). Results of the administration of selected microbial strains ranged from a lack of effect on systemic inflammation (195), no effect on immune activation biomarkers (196, 197) through reduced systemic inflammation markers, but not microbial translocation markers (LPS or sCD14) (198), to a significant decrease of enterocyte apoptosis and increase in Th17 cells in the gut (199). The role of luminal vesicle cargo of microbiome-stabilizing molecules (miRNA and defensins) as potential therapeutic tools to control the growth of pathobionts is emerging, given the development of technologies allowing scalable manufacturing and delivery systems (172). (3) Macrophages are key drivers of the wound-healing process in the intestinal mucosa during the non-pathogenic course of SIV infection in the natural host (156) and participants of the wound-healing process in various disease phenotypes stemming from excessive or insufficient healing (aberrant aging, diabetes, and fibrosis) (200). Therefore, they can provide a novel therapeutic target for promoting wound-healing disorders (151, 200). (4) Downregulation or blockade of SIV/HIV co-receptors. An allogenic CCR5-deficient bone marrow transplant carrying a homozygous deletion (*CCR5* Δ32/Δ32) led to sustained HIV-1 disease remission (201, 202). However, another leukemia patient who received a stem cell transplant from a CCR5-deficient donor showed that post transplant the HIV virus changed tropism from CCR5 toward an alternative co-receptor, CXCR4 (203). Targeting the interaction of CCR5 with HIV led to development of CCR5-blocking agents that prevented virus entry. Maraviroc (MVC) is a non-peptidic small molecule that prevents viral envelope binding (204, 205). Leronlimab, a CCR5-specific antibody (206), effectively increases CCR5^+^ CD4^+^ T cells in circulation in infected humans and macaques (207), suppresses the virus in humans chronically infected with CCR5-tropic HIV-1 and RMs acutely infected with CCR5-tropic SHIV (208), and protects RMs from mucosal SHIV acquisition (209). Downregulation of CD4, which is a natural process in the maturation of CD4^+^ T cells into CD4^neg^ CD8aa^+^ T-cells in non-progressing hosts and does not seem to lead to any pathologies, appears to be a potential approach to effectively prevent cell infection regardless of co-receptor usage (178). (5) SIV-related epigenetic modifications in genes associated with immunoregulation and tissue integrity may constitute novel candidates for immunotherapies (192). (6) PD-1-targeting/related therapies for controlling virus in lymphoid tissues (182). Overexpression of programmed death-1 (PD-1) is characteristic of HIV/SIV-induced exhaustion of the immune system (CD4^+^ and CD8^+^ T cells) (210). PD-1 blockage was leveraged to reduce viral reservoirs in lymphoid tissues and specifically redirect NK cells to the follicles. It increased the therapeutic benefits of SIV vaccine in pathogenic SIV infection in macaques by enhancing the function and follicular homing of vaccine-induced CD8^+^ T cells and decreasing viral reservoirs in lymphoid tissue (211). The high expression of PD-1 in the follicular helper CD4^+^ T cells was harnessed to direct NK-cells expressing a chimeric antigen receptor (CAR) to the follicles and selectively deplete of PD-1^high^ target cells (212).

The last two decades of studies investigating SIV infections in their natural hosts have proven to be critical for driving our understanding of the pathogenicity of SIV infection. Current research points towards therapeutic avenues for controlling the deleterious consequences of pathogenic infections.

## Author contributions

AJ, CA, and IP designed, wrote, and edited the manuscript. All authors contributed to the article and approved the submitted version.
